# Supplementary material for: Evaluating long-term stool preservation methods for maximizing the recovery of viable human fecal microbiota
Source: Gut Microbes Rep. 2025 Dec 7;2(1):2594958. doi: 10.1080/29933935.2025.2594958 (PMC12940166; doi:10.1080/29933935.2025.2594958)
Supplement: Supplementary material — TeoLavrinienko_Supplement [file KGMR_A_2594958_SM8360.docx]

**Supplementary material**

**Title**

Evaluating long-term stool preservation methods for maximizing the recovery of viable human fecal microbiota

**Running title:** Validation of human stool microbiota cryopreservation

**Authors**

Youzheng Teo^1^*, Anton Lavrinienko^2^*, Diana Albertos Torres^3^, Paul Tetteh Asare^1^, Antonia Ruder^2^, Maria Gloria Dominguez-Bello^4,5,6^, Adrian Egli^3^, Nicholas A. Bokulich^2†^, Pascale Vonaesch^1†#^

Notes: *co-first authors, ^†^co-last authors, ^#^corresponding author

Youzheng Teo and Anton Lavrinienko contributed equally to this work.

**Affiliations**

^1^ Department of Fundamental Microbiology, University of Lausanne, Switzerland

^2^ Department of Health Sciences and Technology, ETH Zurich, Switzerland

^3^ Institute of Medical Microbiology, University of Zurich, Switzerland

^4^ Department of Biochemistry and Microbiology, Rutgers University, USA

^5^ Department of Anthropology, Rutgers University, USA

^6^ Humans and the Microbiome Program, Canadian Institute for Advanced

Research, Toronto, ON M5G 1M1, Canada

**Corresponding author:** Pascale Vonaesch: pascale.vonaesch@unil.ch

**Keywords:** human microbiota, cryopreservation, cultivation, stool biobanking, long-term storage

**Supplementary Tables**

**Table S1.** Differences in the human stool microbiota alpha diversity among host age groups.

**Table S2.** Differences in the human stool microbiota alpha diversity between directly sequenced and cultured samples across different host age groups.

**Table S3.** Differences in the human stool microbiota alpha diversity according to sample delivery method and different sample processing strategies.

**Table S4.** Differences in the human stool microbiota alpha diversity across experimental conditions and cryopreservation treatments.

**Table S5.** List of bacterial genera sorted according to their relative abundance and overlap across experimental conditions and cryopreservation treatments.

**Table S6.** Differences in the human stool microbiota alpha diversity across sample storage conditions (-80°C freezer or liquid nitrogen) and processing strategies.

**Table S7.** Differences in the human stool microbiota beta diversity across experimental conditions and cryopreservation treatments.

**Table S8.** Differences in the human stool microbiota beta diversity across sample storage conditions (-80°C freezer or liquid nitrogen) and processing strategies.

**Table S9.** List of differentially abundant bacterial genera between different cryopreservation treatments and directly cultured samples within each host age group.

**Supplementary Figures**

**
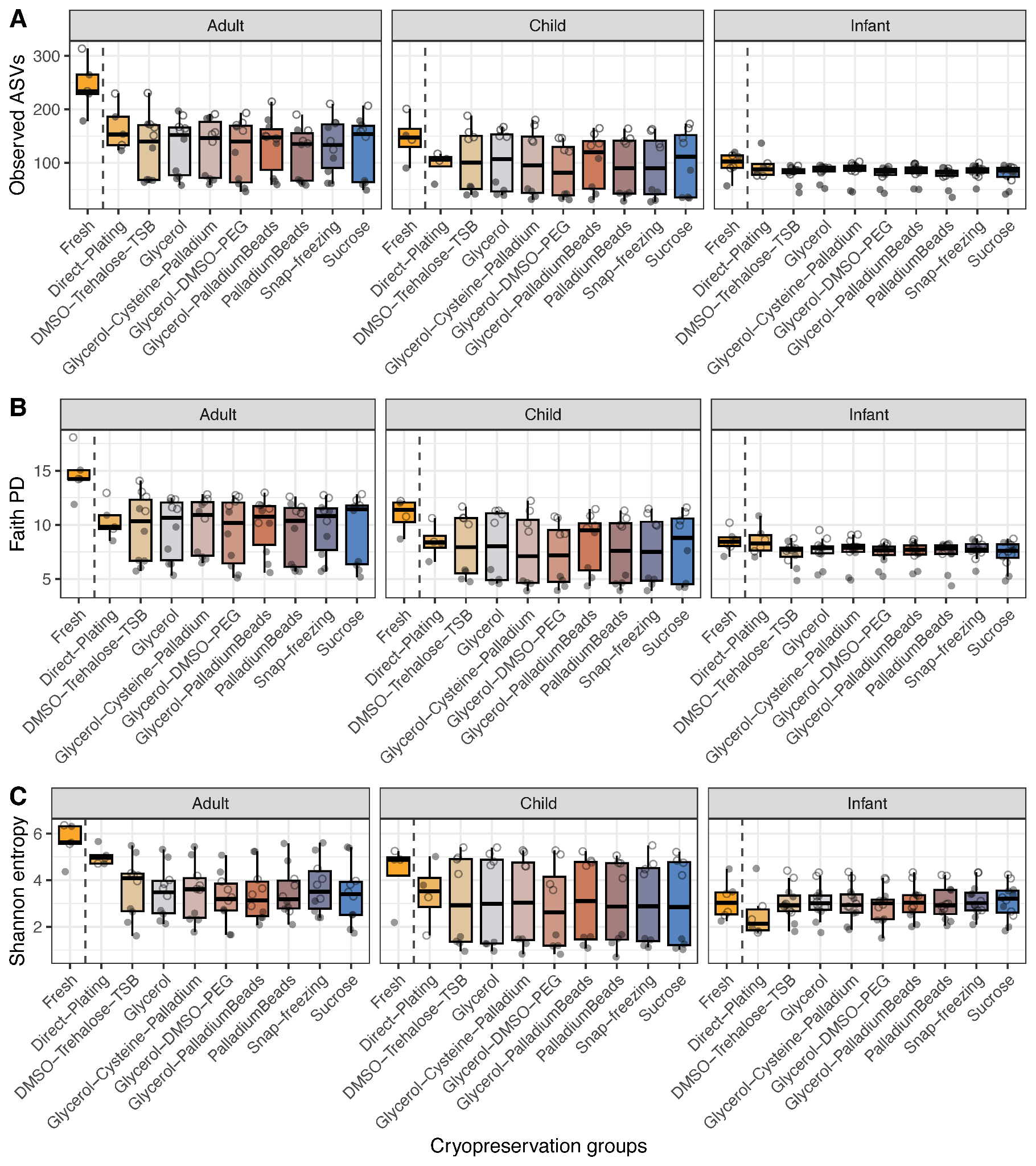
**

**Figure S1. Differences in the human fecal microbiota diversity across experimental conditions and host age groups.** Box-and-whisker plots represent the median and interquartile range of alpha diversity based on the (A) observed ASVs, (B) Faith's phylogenetic diversity (PD), and (C) Shannon entropy across different experimental conditions and cryopreservation treatments. The plot is faceted to display alpha diversity data by host age group (i.e., adult, child, infant). Each point represents a single sample, while shape indicates the sample delivery method: shipped via overnight post (closed points) or collected in person (open points). Dashed line separates directly sequenced samples from plated samples.


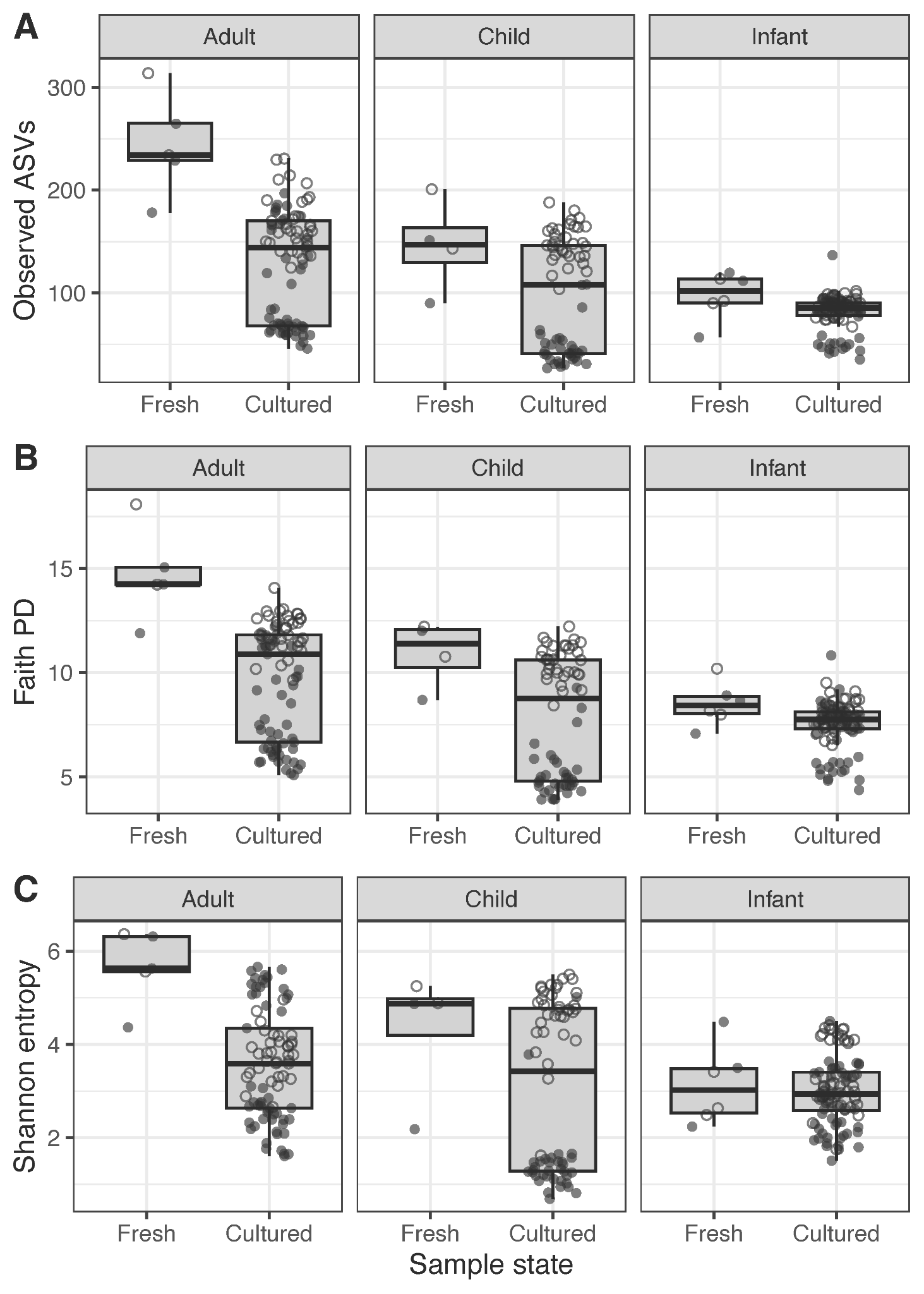


**Figure S2. Differences in the human fecal microbiota diversity between directly sequenced and plated samples.** Box-and-whisker plots represent the median and interquartile range of alpha diversity based on the (A) observed ASVs, (B) Faith's phylogenetic diversity (PD), and (C) Shannon entropy between directly sequenced (fresh) and plated (cultured) samples. The plot is faceted to display alpha diversity data by host age group (i.e., adult, child, infant). Each point represents a single sample, while shape indicates the sample delivery method: shipped via overnight post (closed points) or collected in person (open points).


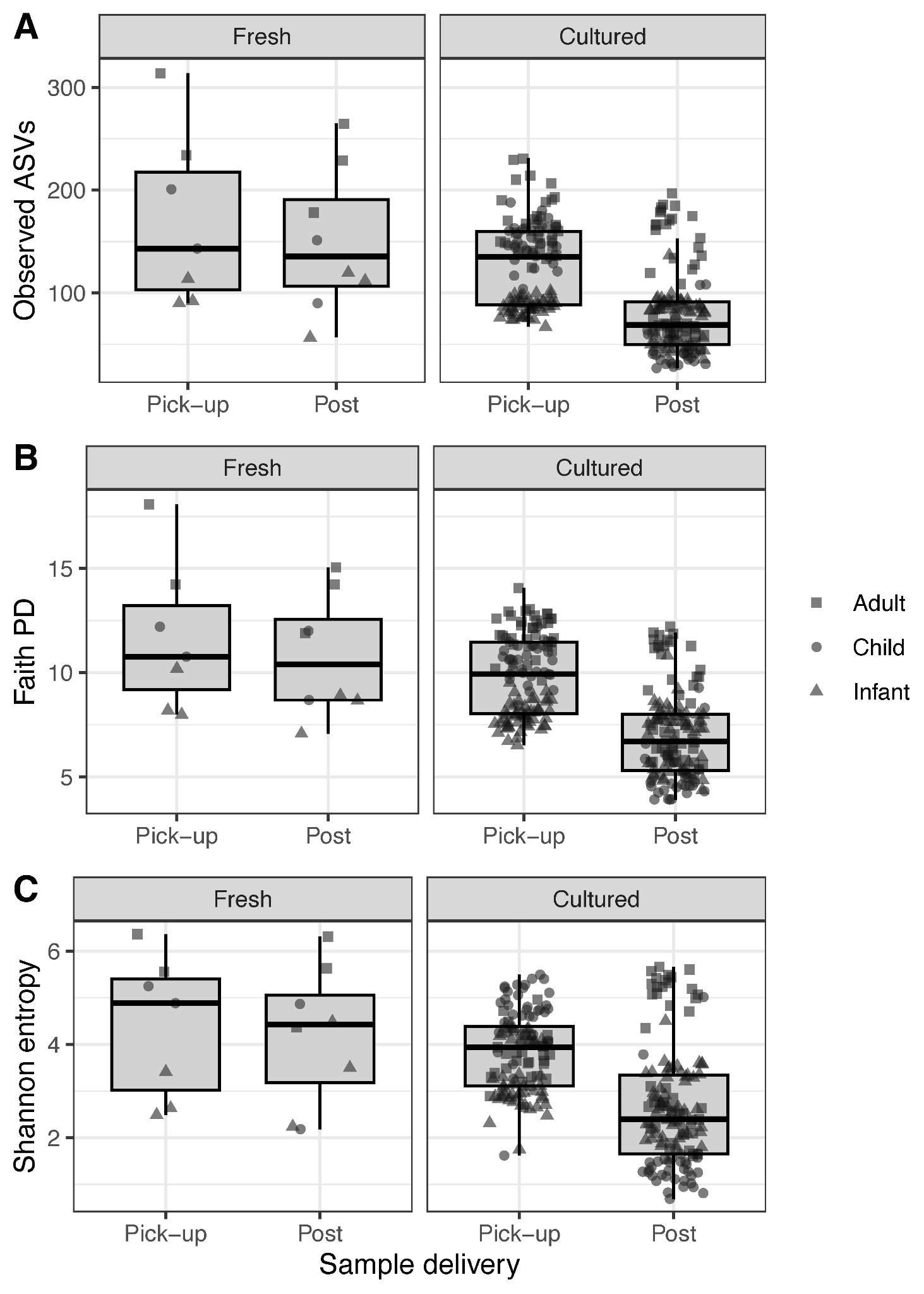


**Figure S3. Differences in the human fecal microbiota diversity between sample delivery methods.** Box-and-whisker plots represent the median and interquartile range of alpha diversity based on the (A) observed ASVs, (B) Faith's phylogenetic diversity (PD), and (C) Shannon entropy between samples collected in person (pick-up) and shipped via overnight post (post). The plot is faceted to display alpha diversity data by sample state: directly sequenced (fresh) and plated (cultured) sample groups. Each point represents a single sample, while shape indicates the host age group (i.e., squares for adults, circles for children, and triangles for infant donors).
